# Alcohol Impairs Immunometabolism and Promotes Naïve T Cell Differentiation to Pro-Inflammatory Th1 CD4^+^ T Cells

**DOI:** 10.3389/fimmu.2022.839390

**Published:** 2022-05-12

**Authors:** Patrick M. McTernan, Danielle E. Levitt, David A. Welsh, Liz Simon, Robert W. Siggins, Patricia E. Molina

**Affiliations:** ^1^Department of Physiology, Louisiana State University Health Sciences Center, New Orleans, LA, United States; ^2^Comprehensive Alcohol-HIV/AIDS Research Center, Louisiana State University Health Sciences Center, New Orleans, LA, United States; ^3^Department of Medicine, Section of Pulmonary/Critical Care Medicine, Louisiana State University Health Sciences Center, New Orleans, LA, United States

**Keywords:** immunometabolism, alcohol, differentiation, mitochondria, glycolysis, CD4^+^ T cell

## Abstract

CD4^+^ T cell differentiation to pro-inflammatory and immunosuppressive subsets depends on immunometabolism. Pro-inflammatory CD4^+^ subsets rely on glycolysis, while immunosuppressive Treg cells require functional mitochondria for their differentiation and function. Previous pre-clinical studies have shown that ethanol (EtOH) administration increases pro-inflammatory CD4^+^ T cell subsets; whether this shift in immunophenotype is linked to alterations in CD4^+^ T cell metabolism had not been previously examined. The objective of this study was to determine whether ethanol alters CD4^+^ immunometabolism, and whether this affects CD4^+^ T cell differentiation. Naïve human CD4^+^ T cells were plated on anti-CD3 coated plates with soluble anti-CD28, and differentiated with IL-12 in the presence of ethanol (0 and 50 mM) for 3 days. Both Tbet-expressing (Th1) and FOXP3-expressing (Treg) CD4^+^ T cells increased after differentiation. Ethanol dysregulated CD4^+^ T cell differentiation by increasing Th1 and decreasing Treg CD4^+^ T cell subsets. Ethanol increased glycolysis and impaired oxidative phosphorylation in differentiated CD4^+^ T cells. Moreover, the glycolytic inhibitor 2-deoxyglucose (2-DG) prevented the ethanol-mediated increase in Tbet-expressing CD4^+^ T cells but did not attenuate the decrease in FOXP3 expression in differentiated CD4^+^ T cells. Ethanol increased Treg mitochondrial volume and altered expression of genes implicated in mitophagy and autophagosome formation (*PINK1* and *ATG7)*. These results suggest that ethanol impairs CD4^+^ T cell immunometabolism and disrupts mitochondrial repair processes as it promotes CD4^+^ T cell differentiation to a pro-inflammatory phenotype.

## Introduction

At-risk alcohol use is the most common and costly form of substance use in the United States (US), and approximately 29% of the US adult population meets diagnostic criteria for alcohol use disorder (AUD) (1–4). At-risk alcohol use increases persistent systemic inflammation, which is one of the major mechanisms linked to alcohol-associated end-organ injury (5). Clinical studies show that alcohol-induced systemic inflammation leads to increased susceptibility to infections, impaired bacterial clearance and increased disease burden (5–9). Preclinical studies have shown that chronic ethanol administration increases activated, proliferating CD4^+^ T cells, and dysregulates effector cell differentiation by increasing pro-inflammatory Th17 CD4^+^ T cells in the gut while increasing the ratio of T-Box Expressed in T cells (Tbet)-expressing (Th1) to Forkhead Box P3 (FOXP3)-expressing (Treg) CD4^+^ T cells in the colon (10–16). These changes to immune cell activation and differentiation promote a pro-inflammatory environment that can increase the risk of developing autoimmune disorders (17) and susceptibility to infections, including human immunodeficiency virus (HIV), which targets activated CD4^+^ T cells (18, 19).

The adaptive immune system is critical for immunological memory and a sustained immune response to antigenic and inflammatory signals, and T cells are important in this process. T cells are classified as CD4^+^ and CD8^+^ based on the expression of an accessory glycoprotein co-receptor, which is responsible for their interaction with major histocompatibility complex (MHC) class II or class I molecules, respectively (20, 21). While CD8^+^ T cells are important in attacking infected or malignant cells, CD4^+^ T cells are responsible for the recruitment of cytotoxic T cells to sites of infection, mediating the humoral antibody response, and regulating innate and acquired immune cell activation and proliferation.

Chronic ethanol administration impairs normal metabolism in tissues, such as the liver, including glycolysis and mitochondrial electron transport chain (ETC) complexes (22–24). This impairment of glycolysis within the liver occurs, in part, due to an increase in the NADH/NAD^+^ ratio, which depletes free NAD^+^ cofactor availability for glycolytic enzymes, such as glyceraldehyde 3-phosphate dehydrogenase (25). Ethanol also decreases myoblast (muscle stem cells) glycolytic function and leads to decreased differentiation (26). Further, ethanol increases reactive oxygen species (ROS) formation in mitochondria, which can damage mitochondrial DNA and ETC proteins, leading to impaired mitochondrial function and cell death (24, 27). Though the ethanol-mediated changes to tissue metabolism are well characterized, ethanol changes to immunometabolism have not been previously investigated.

CD4^+^ T cells rely on both glycolysis and oxidative phosphorylation to support energy requirements of immune responses (28). Naïve CD4^+^ T cells exist in a quiescent state and rely on fatty acid oxidation as their primary energy source. Upon binding of the T cell receptor (TCR), T cells are activated, and metabolism shifts from fatty acid oxidation to aerobic glycolysis, a process necessary to meet the energetic demands of the proliferating, activated T cells (28). After activation of naïve CD4^+^ T cells, transcription factor networks direct differentiation, and the expression of these networks depends on distinct metabolic pathways (29–31). Expression of master transcription factors *Tbet* (Th1), GATA Binding Protein 3 (*GATA3*; Th2), and Retinoic Acid-Related Orphan Receptor Gamma T (*RORγt*; Th17) all depend on glycolytic pathway activation, specifically Mammalian Target of Rapamycin Complex 1 (MTORC1), MTORC2, and Hypoxia Inducible Factor 1 subunit alpha (HIF-1α) signaling, respectively, for their expression. *FOXP3 (*Treg) expression relies on oxidative phosphorylation and adenosine monophosphate-activated protein kinase (AMPK) inhibition, which is important for the promotion of mitochondrial biogenesis (32). Impairment of either of these bioenergetic pathways can dysregulate normal CD4^+^ T cell differentiation. Studies using 2-deoxyglucose (2-DG), an inhibitor of glycolysis, or rapamycin, an inhibitor of mTOR, shifted CD4^+^ differentiation from Th1, Th2, and Th17, towards a Treg phenotype (33–35). Moreover, activating mTOR^-/-^ T cells only generates Treg cells and no other effector T cells (36). On the other hand, Treg differentiation can be impaired by using etomoxir, an inhibitor of complex I in the electron transport chain (ETC) (37).

Mitochondria play a crucial role in cellular energy metabolism and Treg differentiation (29, 32). Both preclinical and clinical studies have demonstrated that ethanol alters mitochondrial function by impairing mitochondrial biogenesis, increasing mitochondrial DNA damage, and increasing oxidative stress (38–40). Further, ethanol dysregulates gene expression implicated in mitohormesis, a response to stress to restore health and viability of mitochondria, and induces mitochondrial DNA (mtDNA) damage within alveolar macrophages (41, 42). Myoblasts isolated from persons living with HIV (PLWH) with high alcohol use disorder identification test (AUDIT) scores showed increased mitochondrial content and decreased bioenergetic health index compared to PLWH with low AUDIT scores (43). This mitochondrial dysfunction is speculated to be due to ethanol-mediated mitochondrial damage and defective mitophagy (44) as shown in previous studies where ethanol decreased expression of genes important to mitophagy, including *PINK1* and *PARKIN* (44). These ethanol-mediated impairments of mitochondrial homeostatic processes could significantly impact the capacity for Treg differentiation.

This study tested the hypothesis that ethanol impairs bioenergetics of CD4^+^ T cells and dysregulates differentiation. Our results indicate that ethanol promoted differentiation of pro-inflammatory Th1 CD4^+^ T cells by increasing glycolysis, and decreased immunosuppressive Treg CD4^+^ T cell differentiation by impairing mitochondrial function. To date, this is the first report to investigate how ethanol regulates T cell immunometabolism and alter the fate of cell differentiation.

## Materials and Methods

### *In Vitro* Activation and Differentiation of CD4^+^ T Cells and Ethanol Treatments

Peripheral blood mononuclear cells (PBMCs) were isolated from blood bank donor buffy coats using a Ficoll-Paque gradient (Cytiva, Marlborough, MA) and frozen in 10% DMSO (ThermoFisher, Waltham, MA) in fetal bovine serum (FBS) at -80° C (45). PBMCs were thawed and washed twice using growth media [RPMI 1640 supplemented with 10% FBS, 3 mM L-glutamine, and 100 U/ml penicillin/streptomycin (Gibco, Waltham, MA)] and cultured overnight at 37°C, 5% CO_2_ and 100% relative humidity. Naïve CD4^+^ T cells were sorted using the human Naïve CD4^+^ T cell Isolation Kit II and LS MACS sorting columns (Miltenyi Biotec, Auburn, CA) following the manufacturer’s protocol. Naïve CD4^+^ T cells were then activated and differentiated by plating at a density of 2 x 10^5^ cells in 100 ul (2x10^6^/ml) per well on coated anti-CD3 (5 μg/ml; catalog # 16-0037-81; ThermoFisher) 96 well flat-bottom plates (Costar, Washington DC, MD) in the presence of soluble anti-CD28 (2.5 μg/ml; catalog # 16-0289-81; ThermoFisher) and IL-12 (25 ng/ml; catalog #573002; Biolegend, San Diego, CA) for 3 days in 37°C incubators with 5% CO_2_ with and without 50 mM ethanol. IL-12 was added to growth media (ThermoFisher) to promote both Th1 and Treg differentiation (DIFF). There was no difference in promotion of FOXP3 expression with either IL-12 or TGF-beta and IL-2 stimulated CD4^+^ T cells (**Supplementary Figure 1**). Growth media with no IL-12, was referred to as control media (Act). Naïve CD4^+^ T cells that were not activated and differentiated were plated (density of 2x10^6^/ml per well) in growth media without anti-CD3 and anti-CD28. Water pans containing 75 mM ethanol were changed daily to maintain the 50 mM ethanol concentration in the incubator atmosphere as previously described (26). Both 25 mM and 50 mM ethanol were used in preliminary studies, but 25 mM had no significant effect on CD4^+^ T cell differentiation (data not shown), hence all subsequent experiments were performed with 50 mM. Experimental groups for this study are defined as:

- Naïve – naïve CD4^+^ T cells that are not activated and differentiated or treated with ethanol.
- EtOH – naïve CD4^+^ T cells that are not activated and differentiated but are treated with 50 mM ethanol.
- DIFF – naïve CD4^+^ T cells that are activated, using anti-CD3 and anti-CD28, and differentiated, using IL-12, and not treated with 50 mM ethanol.
- DIFF + EtOH – naïve CD4^+^ T cells that are activated, using anti-CD3 and anti-CD28, and differentiated, using IL-12, and treated with 50 mM ethanol.

### Flow Cytometry

CD4^+^ T cells were immunostained with antibodies purchased from Biolegend unless otherwise specified using the following panel (CD4^+^ differentiation panel): CD4-APC (clone: A161A1), CD45RA-BV570 (clone: HI100), CD38-AF700 (clone: HIT2), CD25-BV650 (clone: BC96), CD3-PE-Cy7 (clone: OKT3), CD28-BV605 (clone: CD28.2), FOXP3-PE-CY5 (clone: PCH101) (ThermoFisher, MA, USA), Tbet-PE (clone: 4B10), GATA3-BV421 (clone: 16E10A23), RORγt -PE-CF594 (clone: Q31-378) (BD Biosciences, Franklin Lake, NJ), Live/Dead – eFluor780 (Invitrogen, Waltham, MA). The staining was performed in two steps. The gating strategy for the CD4 differentiation panel is shown as **Supplementary Figure 2**. Cells were incubated with antibodies (1 μg) targeting CD4, CD45RA, CD38, CD25, and Live/Dead at 4°C for 30 minutes. Cells were then fixed for 30 minutes at room temperature in the dark using the Foxp3/Transcription Factor Staining Buffer Set (catalog #: 00-5523-00; ThermoFisher) according to the manufacturer’s protocol. Following permeabilization, cells were stained with antibodies targeting CD3, CD28, FOXP3, Tbet, GATA3, and RORγt at 4°C overnight for 16-18 hours. GLUT1 expression was assessed using a separate panel, and included CD3, CD4, Tbet, and FOXP3 from the CD4^+^ differentiation panel. GLUT1 (R&D Systems; clone: MAB1418) was conjugated with FITC in the laboratory using the Lighting-Link FITC conjugation kit (catalog #: 707-0010; Novus Biologicals, CO, USA). Samples were analyzed using an 18-color BD LSRII flow cytometer and FACSDiva software (ver. 8.0.1, Becton, Dickinson, Franklin Lakes, NJ). Flow cytometry gates were defined based on fluorescence minus one (FMO) controls.

### Sorting of CD25^+^-Expressing CD4*^+^* T Cells

The differentiated CD4^+^ T cells were sorted to Tbet- and FOXP3-enriched CD4^+^ T cell subsets using the human CD4^+^CD25^+^ regulatory T cell isolation kit (catalog #: 130-091-301; Miltenyi Biotec, Auburn, CA). After sorting, flow cytometry, using the CD4 Differentiation Panel, confirmed that 29% of CD4^+^CD25^+^ T cells expressed FOXP3 and 21% expressed Tbet. Flow through containing CD4^+^CD25^-^ CD4^+^ T cells were enriched in Tbet-expressing CD4^+^ T cells (18% Tbet and 4% FOXP3; **Supplementary Figure 3**). These sorted populations were defined as either CD4+CD25+ “Enriched Treg” or CD4+CD25- “Enriched Th1”.

### Mitochondrial Function

Mitochondrial oxygen consumption rate (OCR) was measured using a Mito Stress Test (31) and Seahorse XFe96 technology (Agilent Technologies, Santa Clara, CA). Naïve and IL-12-differentiated CD4^+^ T cells were seeded on Cell-Tak (Corning, Corning, NY)-coated 96-well Seahorse plates in triplicate at a density of 3 x 10^5^ cells/well within 200 ul, at a concentration of 1.5 million cells/ml, and maintained under standard cell culture conditions (5% CO_2_, 37^0^C). Cells were incubated in XF Assay Medium (pH 7.4) with sodium pyruvate (1 mM), L-glutamine (2 mM), and glucose (10 mM) at 37°C without CO_2_ for 1 hr before measuring T cell OCR with an XFe96 Extracellular Flux Analyzer (Agilent Technologies, Santa Clara, CA) according to manufacturer’s instructions. Respiratory parameters were assessed by the sequential addition of oligomycin (1 μM), carbonyl cyanide‐p‐trifluoromethoxyphenylhydrazone (FCCP; 2 μM), and rotenone/antimycin A (0.5 μM). OCR measurements were normalized to cell count obtained by staining nuclei with Hoescht dye (2 μM; ThermoFisher Scientific) and visualizing on a BioTek Cytation 1 cell imaging multi-mode reader (BioTek, Winooski, VT).

### Glycolytic Function

A Glycolysis Stress Test (31) was performed in naïve and IL-12-differentiated CD4^+^ T cells seeded as described above. Cells were incubated at 37°C without CO_2_ for 1 hr in glucose-free XF Assay Medium (pH 7.4) with L-glutamine (2 mM) before measuring CD4^+^ T cell extracellular acidification rate (ECAR) at baseline and after the sequential addition of glucose (10 mM), oligomycin (1 μM), and 2-deoxyglucose (2-DG; 50 mM). ECAR measurements were normalized to cell count as described for the Mito Stress Test.

### Mitochondrial Content

T cell mitochondrial content was quantified with MitoTracker Deep Red FM probes (Thermofisher) (46, 47). Naïve and IL-12-differentiated CD4^+^ T cells were incubated in growth media containing MitoTracker Deep Red FM (17 nM) under standard cell culture conditions for one hour. An unstained control sample was simultaneously incubated in growth media without MitoTracker. Cells were pelleted, washed with phosphate-buffered saline (PBS; Gibco) and fixed for flow cytometry using the PerFix-nc Kit (Beckman Coulter, Brea, CA) according to manufacturer instructions. Samples were analyzed on a BD FACSCanto II flow cytometer (Becton, Dickinson and Company). The gating strategy for the Mitotracker Deep Red assay is shown as **Supplementary Figure 4**. Mean fluorescent intensities (MFI) were calculated using BD FACSDiva software (ver 8.0.1).

### Glucose Uptake

Glucose uptake was measured with 2-Deoxy-2-[(7-nitro-2,1,3-benzoxadiazol-4-yl) amino]-D-glucose (2-NBDG) (Sigma-Aldrich, St. Louis USA). Briefly, CD4^+^ T cells were washed with PBS and incubated in glucose-free RPMI 1640 supplemented with 10% FBS, 3 mM L-glutamine, and 100 U/ml penicillin/streptomycin (Gibco, Waltham, MA) for 1 hour. After incubation, 2-NBDG was added to culture wells to a final concentration of 150 μM for 30 minutes at 37°C and 5% CO_2_. CD4^+^ T cells were washed twice with PBS and analyzed using the 533 nm wavelength channel on a BD FACSCanto II flow cytometer (Becton, Dickinson and Company). The gating strategy for the 2-NBDG uptake assay is shown as **Supplementary Figure 5**. Mean fluorescent intensities (MFI) were calculated using BD DIVA software (ver 8.0.1).

### 2-Deoxyglucose Treatment

Naïve CD4^+^ T cells were activated and allowed to differentiate using the *in vitro* differentiation protocol above, and on day 2 of the 3-day culture, 2-DG (0.5 mM) was added for 24 h. After incubation with 2-DG, cells were stained and assessed by flow cytometry using the CD4^+^ differentiation panel as described above.

### Expression of Genes Implicated in Mitochondrial Function

Total RNA was extracted from naïve and IL-12-differentiated CD4^+^ T cells after 3 days in the presence and absence of 50 mM ethanol using the miRNeasy Mini Kit (Qiagen, Valencia, CA) according to manufacturer’s instructions. cDNA was synthesized using the Qiagen RT^2^ First Strand synthesis kit (Qiagen, Valencia, CA) and qPCR performed using custom RT^2^ qPCR profiler arrays (Qiagen: CAPA9632-12:CLAH42284). qPCR reactions were carried out in duplicate using a CFX96 thermal cycler (Bio-Rad, Hercules, CA) with Beta-2-Microglobulin (*B2M*) as the endogenous control. Data were then analyzed using the 2^-ΔΔCt^ method. All genes analyzed are summarized in **Table 1**.

**Table 1:** List of gene targets analyzed by RT^2^ qPCR profiler arrays.

Gene Symbol	Gene Targets	
**Mitochondrial Maintenance and Mitophagy Related Genes**		
*PRKN* & *PINK1*	Parkin & PTEN-induced Kinase 1	
*ATG5, ATG7*, & *ATG13*	Autophagy Related 5, 7, and 13	
*MFN1* & *MFN2*	Mitofusin 1 & 2	
*BNIP3L* & *ULK1*	NIX & Autophagy Activating Kinase 1	
*TFAM*	Transcription Factor A, mitochondrial	
*PPARGC1A* & *PPARGC1B*	PPAR-γ Co-activator α & β	
*MAP1LC3B*	Microtubule Associated Protein 1 Light Chain 3 Beta	
*BECN1*	Beclin 1	
*USP30*	Ubiquitin Specific Peptidase 30	
*OPA1*	OPA1 Mitochondrial Dynamin Like GTPase	
**House Keeping Genes**		
*B2M*	Beta-2-microglobulin	
*RPLPO*	Ribosomal Protein Lateral Stalk Subunit P0	

### Statistical Analysis

Data were checked for assumption of normality using the Shapiro-Wilk test. The alpha level set for all statistical analysis was a p value < 0.05. CD4^+^ differentiation, glycolysis, MitoTracker, and RT^2^ profiler array data were analyzed using a 2 (differentiation) x 2 (ethanol) analysis of variance (ANOVA) with repeated measures on both factors. 2-DG data were analyzed using a 2 (differentiation) x 2 (ethanol) x 2 (2-DG) ANOVA with repeated measures on all factors. When appropriate, *post hoc* pairwise comparisons were conducted and p-values adjusted using Tukey’s HSD for 2-way ANOVA and Sidak for 3-way ANOVA. To analyze the effect of IL-12 on CD4^+^ differentiation, and of ethanol on Tbet-FOXP3-expressing CD4^+^ T cells, paired T tests were performed. Paired T tests were also performed to assess the effect of ethanol on CD4^+^ T cell mitochondrial function and to analyze the MitoTracker data within Treg and Th1 CD4^+^ subsets. The Treg OCR-linked proton leak data did not pass normality or lognormality tests and therefore a Wilcoxon test was used to analyze significance. All analyses except 3-way ANOVA were performed using GraphPad prism 9.2.0 (San Diego, CA). 3-way ANOVA analyses were conducted using SPSS (Version 25, IBM Corporation, Armonk, NY).

## Results

### IL-12 Promotes CD4^+^ T Cell Expression of Tbet

**Figure 1:**
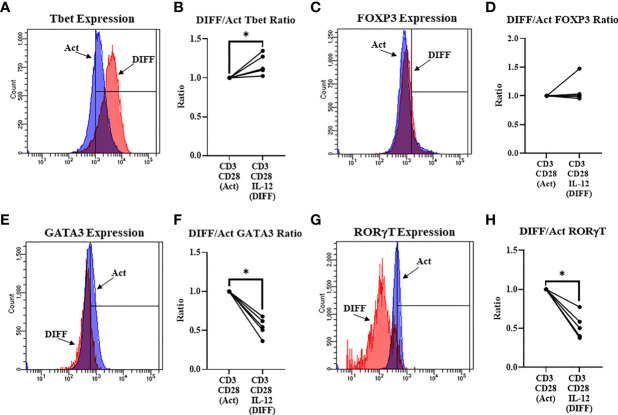
Differentiation (Anti-CD3, Anti-CD28, & IL-12) of naïve CD4^+^ T cells. **(A)** Representative FACS histograms of Tbet expression in Act and DIFF CD4^+^ T cells **(B)** IL-12 increased the ratio of CD4^+^ T cells expressing Tbet. **(C)** Representative FACS histograms of FOXP3 expression in Act and DIFF CD4^+^ T cells. **(D)** IL-12 had no effect on CD4^+^ T cells expressing FOXP3. **(E)** Representative FACS histograms of GATA3 expression in Act and DIFF CD4^+^ T cells. **(F)** IL-12 decreased the ratio of CD4^+^ T cells expressing GATA3. **(G)** Representative FACS histograms of RORgT expression in Act and DIFF CD4^+^ T cells. **(H)** IL-12 decreased the ratio of CD4^+^ T cells expressing RORgT. Act, Activation; DIFF, Differentiation. Data are average values for 3 independent experiments using CD4^+^ T cells from 5 donors. Significant differences (p<0.05) were detected using a paired T-test. *p ≤ 0.05.

IL-12 was used to direct differentiation of CD4^+^ T cells. IL-12 preferentially directed differentiation of Tbet- (Th1; p = 0.006; **Figures 1A, B**) and had no effect on FOXP3- (Treg; **Figures 1C, D**) expressing CD4^+^ T cells, while decreasing the expression of GATA3 (Th2; p < 0.001; **Figures 1E, F**) and RORγt (Th17; p < 0.01; **Figures 1G, H**).

### Alcohol Promotes CD4^+^ T Cell Differentiation Towards a Pro-Inflammatory Phenotype

**Figure 2:**
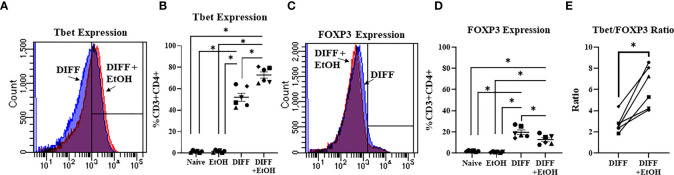
Effect of ethanol on CD4^+^ T cell differentiation. **(A)** Representative FACS histograms of Tbet expression in DIFF and DIFF + EtOH CD4^+^ T cells. **(B)** Ethanol increased percent of CD4^+^ Tbet-expressing cells. **(C)** Representative FACS histograms of FOXP3 expression in DIFF and DIFF + EtOH CD4^+^ T cells. **(D)** Ethanol decreased percent expression of FOXP3 in CD4^+^ T cells. **(E)** Ethanol increased the Tbet/FOXP3 ratio in differentiated CD4^+^ T cells. Naïve = undifferentiated and no ethanol treatment, EtOH = undifferentiated and ethanol-treated, DIFF = differentiated and no ethanol treatment, and DIFF + EtOH = differentiated and ethanol-treated. Data are average values for 3 independent experiments using CD4^+^ T cells from 6 donors expressed as mean ± SEM. Significant differences (p<0.05) were determined by repeated measures 2-way ANOVA (Panels **B, D**) and paired T-tests (Panel **E**).*p ≤ 0.05.

To test the impact of alcohol on CD4^+^ T cell differentiation, naïve CD4^+^ T cells were differentiated in the presence of 50 mM ethanol under Th1-promoting conditions. There was a significant interaction observed between DIFF and EtOH on both Tbet (p < 0.05) and FOXP3-expressing (p < 0.01) CD4^+^ T cells. *Post hoc* pairwise comparisons indicated that differentiation of CD4^+^ T cells significantly increased Tbet-expressing CD4^+^ T cells compared to undifferentiated naïve CD4^+^ T cells (p < 0.0001) and ethanol increased expression of Tbet within differentiated CD4^+^ T cells (p = 0.001; **Figures 2A, B**). Also, *post hoc* pairwise comparisons indicated that differentiation significantly increased FOXP3-expressing CD4^+^ T cells compared to undifferentiated naïve CD4^+^ T cells (p < 0.0001) and ethanol decreased expression of FOXP3 in differentiated CD4^+^ T cells (p = 0.001; **Figures 2C, D**). Ethanol increased the Tbet : FOXP3 ratio (p = 0.004; **Figure 2E**) in differentiated CD4^+^ T cells. No significant differences were detected between undifferentiated Naïve and EtOH-treated groups.

### Alcohol Increases Glycolysis and Glycolytic Capacity in Differentiating CD4^+^ T Cells

**Figure 3:**
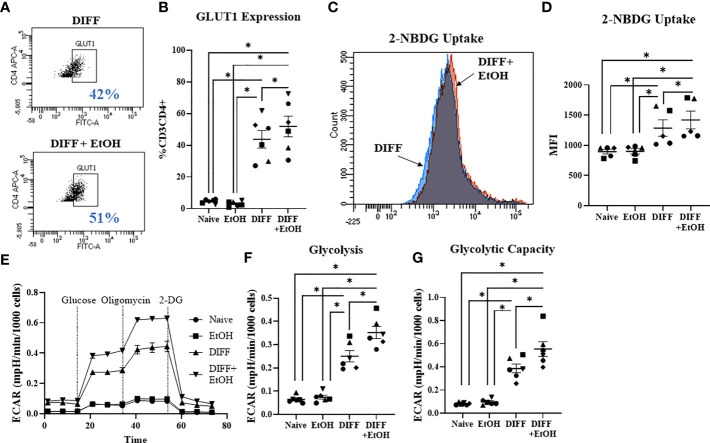
Effect of ethanol on CD4^+^ T cell glucose metabolism. **(A)** Representative FACS plots of GLUT1 expression in DIFF and DIFF + EtOH CD4^+^ T cells. **(B)** Ethanol increased CD4^+^ T cell GLUT1 expression. **(C)** Representative FACS histograms of 2-NBDG uptake in DIFF and DIFF + EtOH CD4^+^ T cells. **(D)** Ethanol increased glucose uptake within differentiated CD4^+^ T cells. **(E)** All experimental groups were analyzed using the Glycolytic Stress test. Ethanol increased glycolysis **(F)** and glycolytic capacity **(G)** within differentiated groups. MFI = mean fluorescent intensity, 2-NBDG = 2-Deoxy-2-[(7-nitro-2,1,3-benzoxadiazol-4-yl)amino]-D-glucose, ECAR = extracellular acidification rate, Naïve = undifferentiated and no ethanol treatment, EtOH = undifferentiated and ethanol-treated, DIFF = differentiated and no ethanol treatment, and DIFF + EtOH = differentiated and ethanol-treated. Data represents average values using CD4^+^ T cells from 6 donors expressed as mean ± SEM. Significant differences (p<0.05) were determined by repeated measures 2-way ANOVA. *p ≤ 0.05.

Glycolysis was assessed to understand if metabolism played a role in the ethanol-mediated increase of Tbet-expressing CD4^+^ T cells. There was a significant interaction observed between DIFF and EtOH on CD4^+^ T cell GLUT1 expression (p < 0.01), 2-NBDG uptake (p < 0.05), glycolysis (p < 0.0001) and glycolytic capacity (p < 0.0001). *Post hoc* pairwise comparisons indicated that differentiation of CD4^+^ T cells significantly increased GLUT1 expression (p < 0.0001; **Figures 3A, B**), glucose uptake (2-NBDG; p = 0.0002; **Figures 3C, D**), glycolysis (p < 0.0001; **Figures 3E, F**) and glycolytic capacity (p < 0.0001; **Figure 3G**) compared to undifferentiated naïve CD4^+^ T cells. Also, *post hoc* pairwise comparisons indicated that ethanol increased GLUT1 expression (p = 0.005; **Figure 3B**), glucose uptake (p = 0.014; **Figure 3D**), glycolysis (p = 0.005; **Figure 3F**) and glycolytic capacity (p < 0.0001; **Figure 3G**) within differentiated CD4^+^ T cells. No significant differences were detected between undifferentiated naïve and naïve EtOH groups.

### Inhibition of Glycolysis With 2-Deoxyglucose (2-DG) Prevents the Alcohol-Mediated Increase in CD4^+^ T Cell Tbet-Expression

**Figure 4:**
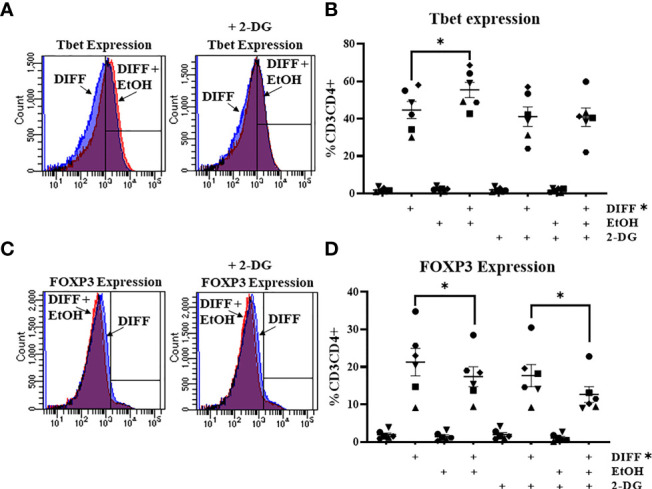
Effect of 2-deoxyglucose (2-DG) on CD4^+^ T cells. **(A)** Representative FACS histograms plots of Tbet expression in DIFF, DIFF + EtOH, and DIFF+ EtOH + 2-DG CD4^+^ T cells. **(B)** The addition of 0.5 mM 2-deoxyglucose (2-DG) prevents the ethanol-mediated increase of Tbet-expressing CD4^+^ T cells. **(C)** Representative FACS histograms plots of FOXP3 expression in DIFF, DIFF + EtOH, and DIFF+ EtOH + 2-DG CD4^+^ T cells. **(D)** The addition of 0.5 mM 2-deoxyglucose (2-DG) did not alter the ethanol-mediated decrease of FOXP3-expressing CD4^+^ T cells. Naïve = undifferentiated and no ethanol treatment, EtOH = undifferentiated and ethanol-treated, DIFF = differentiated and no ethanol treatment, and DIFF + EtOH = differentiated and ethanol-treated. Data represents average values using CD4^+^ T cells from 6 donors expressed as mean ± SEM. Significant differences (p<0.05) were determined by repeated measures 3-way ANOVA. *Post-hoc* analysis was done using Sidaks multiple comparison test. *p ≤ 0.05.

To further understand the importance of glycolysis on the ethanol-mediated increase of Tbet-expressing CD4^+^ T cells, CD4^+^ T cells were exposed with ethanol in the presence of the glycolytic inhibitor 2-deoxyglucose (2-DG). The was a significant interaction observed between DIFF, EtOH, and 2-DG on Tbet-expressing CD4^+^ T cells (p < 0.05) and a significant interaction observed between DIFF and EtOH on FOXP3-expressing CD4^+^ T cells (p < 0.05). *Post hoc* pairwise comparisons indicated that differentiation increased Tbet (p < 0.0001; **Figures 4A, B**) and FOXP3-expressing (p < 0.0001; **Figures 4C, D**) CD4^+^ T cells compared to undifferentiated naïve CD4^+^ T cells. Ethanol increased Tbet-expression within differentiated CD4^+^ T cells (p = 0.002; **Figures 4A,B**). 2-DG prevented the ethanol-induced increase in Tbet-expression in differentiated CD4^+^ T cells (**Figures 4A, B**). Ethanol decreased CD4^+^ T cell FOXP3 expression within differentiated CD4^+^ T cells (p = 0.024; **Figures 4C, D**) irrespective of 2-DG treatment (p = 0.012; **Figures 4C, D**). No significant differences were detected between undifferentiated naïve and naïve EtOH groups.

### Alcohol Impaired Differentiated CD4^+^ T Cell Mitochondrial Function

**Figure 5:**
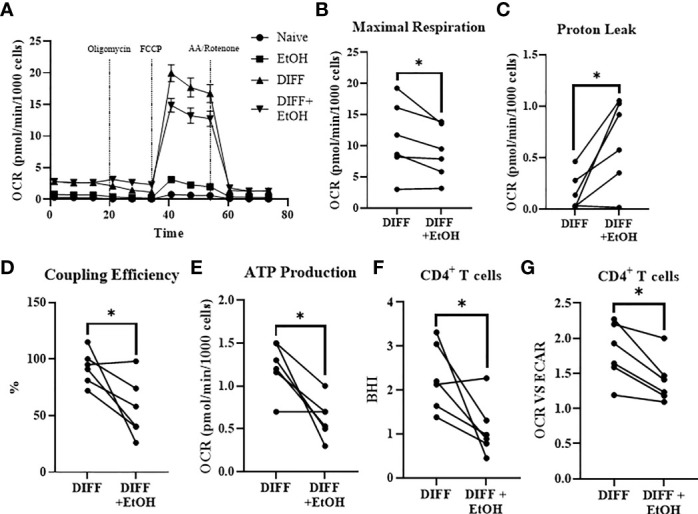
Impact of ethanol on CD4^+^ T cell mitochondrial function. **(A)** All experimental groups were analyzed using the Mitostress test. **(B)** Ethanol decreased maximal respiration. **(C)** Ethanol increased mitochondrial proton leak. **(D)** Ethanol decreased coupling efficiency and **(E)** ATP production. Ethanol decreased **(F)** BHI and **(G)** OCR/ECAR ratio in differentiated CD4^+^ T cells. OCR= oxygen consumption rate; FCCP, Carbonyl cyanide-p-trifluoromethoxyphenylhydrazone; AA, Antimycin; BHI, Bioenergetic Health Index; DIFF, differentiated and no ethanol treatment, and DIFF + EtOH, differentiated and ethanol-treated. Data represents CD4^+^ T cells from 6 donors with lines connecting values of same donors. Significance differences (p ≤ 0.05) were determined by paired T-tests. *p ≤ 0.05.

In order to understand the ethanol-mediated decrease in FOXP3-expressing CD4^+^ T cells, mitochondrial parameters were assessed using the Mitostress test. Ethanol decreased maximal respiration (p = 0.049; **Figures 5A, B**) and increased OCR-linked proton leak within the differentiated CD4^+^ T cells (p = 0.022; **Figure 5C**). Ethanol decreased coupling efficiency (p = 0.036; **Figure 5D**), and OCR-linked ATP production (p = 0.014; **Figure 5E**) within the differentiated CD4^+^ T cells. Ethanol decreased Bioenergetic Health Index (BHI; p = 0.04; **Figure 5F**) and decreased the OCR/ECAR ratio (p = 0.01; **Figure 5G**) within differentiated CD4^+^ T cells.

### Alcohol Impaired CD4^+^CD25+ T Cell Mitochondrial Function

**Figure 6:**
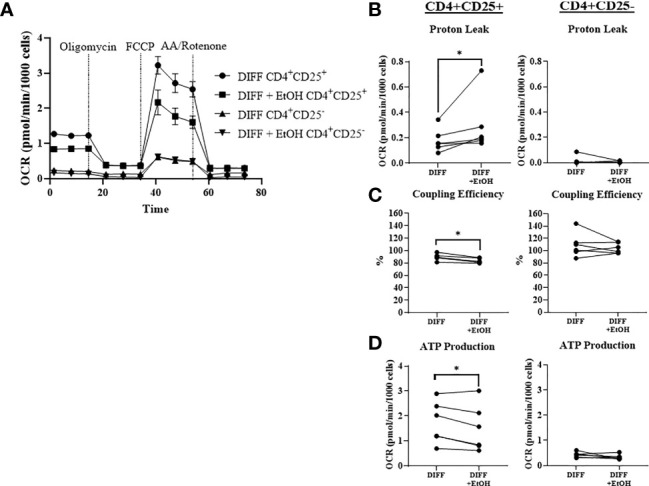
Mitochondrial function within CD4^+^ CD25^+^ (enriched Treg) and CD4^+^ CD25^-^ (enriched Th1) CD4^+^ subsets. **(A)** Enriched Treg and Th1 cells from DIFF and DIFF + EtOH groups were analyzed using Mitostress test. **(B)** Ethanol increased proton leak in enriched Treg, but not enriched Th1 cells. **(C)** Ethanol decreased coupling efficiency in enriched Treg, but not enriched Th1. **(D)** Ethanol decreased ATP production in enriched Treg, but not enriched Th1 cells. OCR= oxygen consumption rate. FCCP = Carbonyl cyanide-p-trifluoromethoxyphenylhydrazone. AA = Antimycin **(A)** DIFF = differentiated cells and no ethanol treatment, and DIFF + EtOH = differentiated and ethanol-treated. Data represents CD4^+^ T cells from 6 donors with lines connecting values of same donors. Significance differences (p ≤ 0.05) were determined by paired T-tests and Wilcoxin test (Treg – proton leak). *p ≤ 0.05.

To further understand the ethanol-mediated decrease in mitochondrial function within CD4^+^ T cells, cells were sorted with the human CD4+CD25+ regulatory T cell isolation kit to enrich Treg (CD4+CD25+ T cells) and negatively select CD4+CD25- Th1-enriched T cells. Ethanol increased OCR-linked proton leak in enriched Treg cells (p = 0.0313; **Figures 6A, B**). Ethanol decreased coupling efficiency (p = 0.0031; **Figure 6C**) and OCR-linked ATP production (p = 0.044; **Figure 6D**) in enriched Treg cells. There were no observed significant effects of ethanol on mitochondrial parameters within enriched Th1 cells. There were no differences for BHI or OCR/ECAR ratio observed between the enriched populations (**Supplementary Figure 6**)

### Alcohol Increased CD4^+^ T Cell Mitochondrial Volume

**Figure 7:**
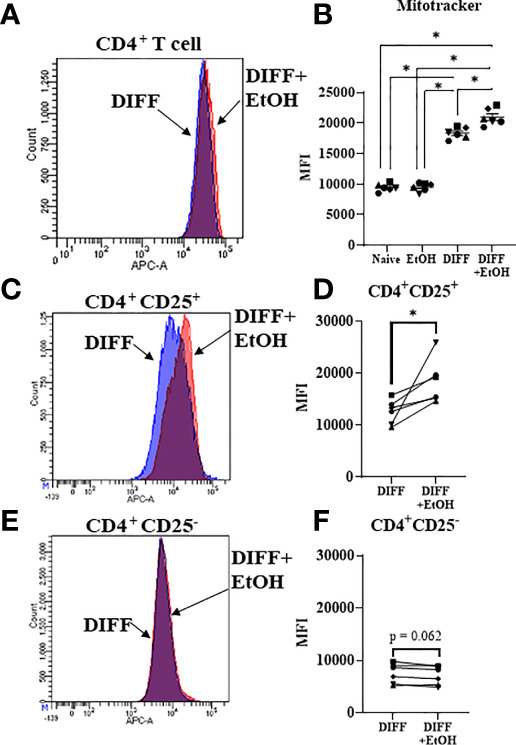
Mitochondrial volume of CD4^+^ T cells. **(A)** Representative FACS histogram of Mitotracker Deep Red staining of DIFF and DIFF + EtOH CD4^+^ T cells. **(B)** Ethanol increased the MFI of Mitotracker Deep Red in differentiated CD4^+^ T cells. **(C)** Representative FACS histogram of Mitotracker Deep Red staining of DIFF and DIFF + EtOH CD4^+^ CD25^+^ (enriched Treg) cells. **(D)** Ethanol increased the MFI of Mitotracker Deep Red in enriched Treg cells. **(E)** Representative FACS histogram of Mitotracker Deep Red staining of DIFF and DIFF + EtOH CD4^+^ CD25^-^ (enriched Th1) cells. **(F)** There were no significant differences in MFI of enriched Th1 cells. MFI = mean fluorescent intensity, Naïve = undifferentiated and no ethanol treatment, EtOH = undifferentiated and ethanol-treated, DIFF = differentiated cells and no ethanol treatment, and DIFF + EtOH = differentiated and ethanol-treated. Data represents average values using PBMCS from 6 donors expressed as mean ± SEM. Significant differences (p<0.05) were determined by repeated measures 2-way ANOVA (Panel **B** )and paired T-tests (Panels **D, F**). *p ≤ 0.05.

Since mitochondrial function was impaired within ethanol-treated CD4^+^ T cells, mitochondrial content was assessed to determine any further potential ethanol-mediated dysfunction. There was a significant interaction observed between DIFF and EtOH on CD4^+^ T cell mitochondrial content (p < 0.01). *Post hoc* pairwise comparisons indicated that differentiation increased mean fluorescence intensity (MFI) of MitoTracker Deep Red within CD4^+^ T cells compared to undifferentiated naïve cells (p < 0.0001; **Figures 7A, B**) and ethanol increased MitoTracker MFI within differentiated CD4^+^ T cells (p = 0.004; **Figures 7A, B**). To determine if both Th1 and Treg cells are contributing to this ethanol-mediated increase in mitochondrial content, enriched CD4^+^ subsets were stained. Ethanol increased MitoTracker MFI in enriched Treg cells (p = 0.038; **Figures 7C, D**), with no observed significant increase in enriched Th1 cells (p = 0.062; **Figures 7E, F**).

### Alcohol and Differentiation Altered CD4^+^ T Cell Mitochondrial Gene Expression

**Figure 8:**
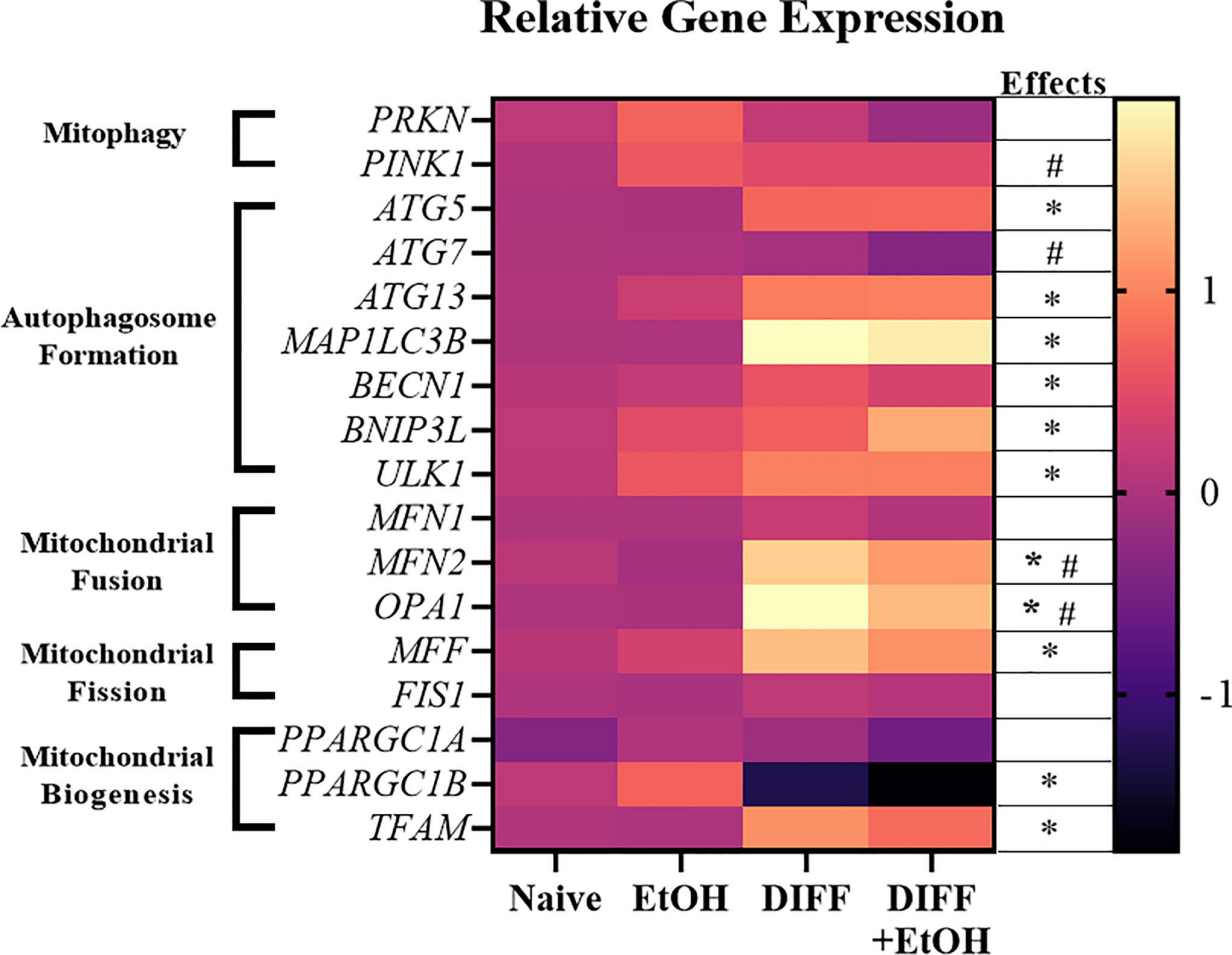
Heat map showing increased and decreased gene expression in response to ethanol or differentiation. Data are normalized as fold change to naïve CD4^+^ T cell gene expression and represents data using PBMCs from 6 matched donors. Naïve = undifferentiated and no ethanol treatment, EtOH = undifferentiated and ethanol-treated, DIFF = differentiated cells and no ethanol treatment, and DIFF + EtOH = differentiated and ethanol-treated. * differentiation main effect (p ≤ 0.05). ^#^ ethanol main effect (p ≤ 0.05) as determined by repeated measures 2-way ANOVA.

**Figure 9:**
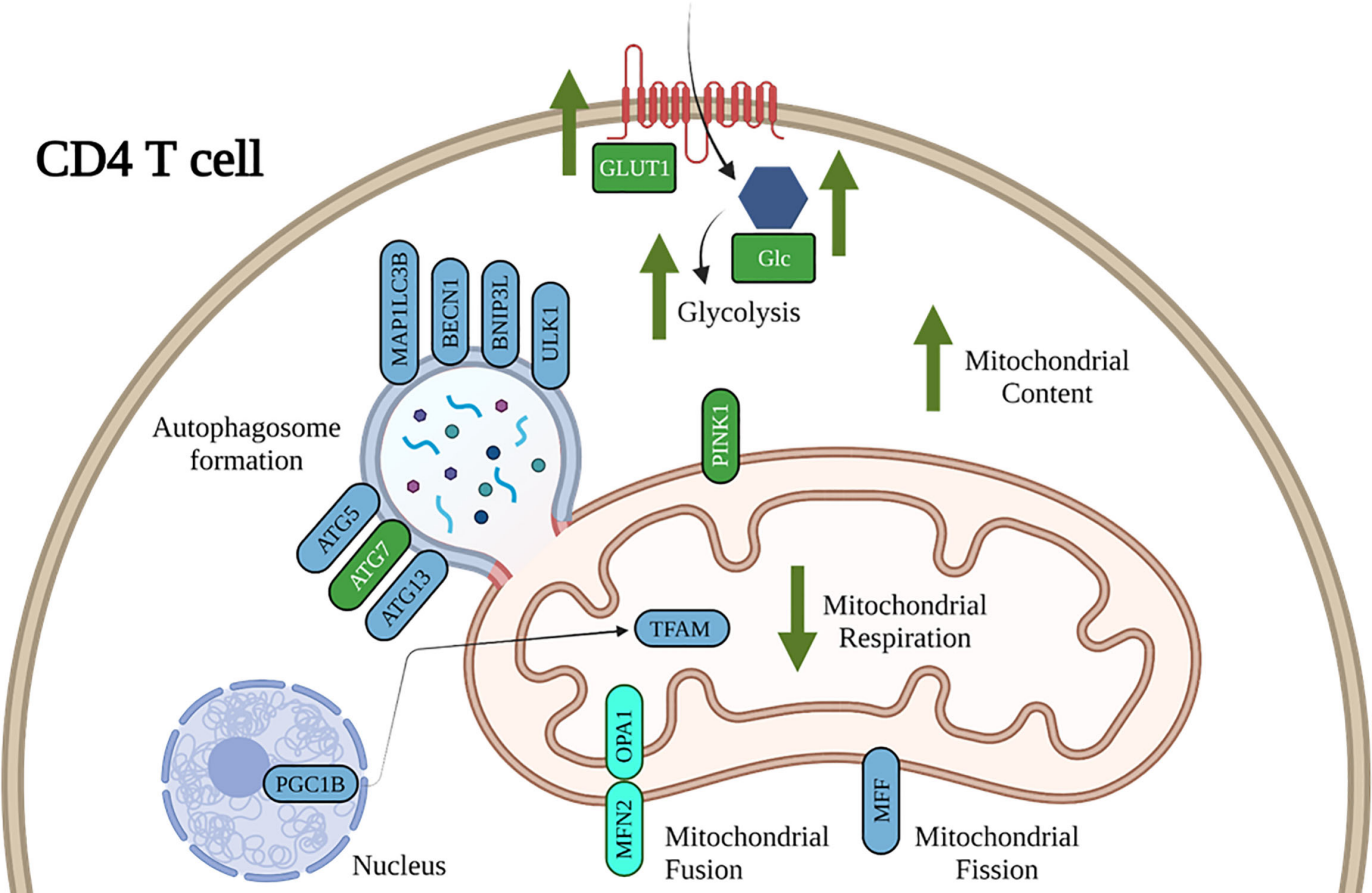
Working model of the impact of ethanol on CD4^+^ T cell energy metabolism and mitochondrial dynamics. Our results show that ethanol increases CD4^+^ T cell glycolytic processes, decreases mitochondrial respiration, and dysregulates gene expression important for CD4^+^ T cell mitochondrial maintenance and mitophagy. Blue denotes gene expression affected by DIFF and green denote gene expression affected by EtOH. Cyan denotes gene expression affected by both DIFF and EtOH.

Gene expression important for mitochondrial repair and mitophagy were assessed to determine the potential impact of ethanol on CD4^+^ T cell mitochondrial homeostasis. There was a main effect of DIFF to increase gene expression important for autophagosome formation (*ATG5*, p = 0.01; *ATG13*, p = 0.04; *MAP1LC3B*, p = 0.01; *BECN1*, p = 0.01; *BNIP3L*, p = 0.05; *ULK1*, p = 0.002; **Figure 8**), mitochondrial biogenesis (*TFAM*, p = 0.03; **Figure 8**), and mitochondrial fission (*MFF*, p = 0.02; **Figure 8**), and decrease *PPARGC1B* (p = 0.005; **Figure 8**) expression. There was a main effect of DIFF and a main effect of EtOH to increase gene expression important for mitochondrial fusion (*MFN2*, DIFF: p = 0.001, EtOH: p = 0.01; *OPA1*: DIFF: p = 0.003, EtOH: p = 0.03; **Figure 8**). There was a main effect of EtOH to increase gene expression important for mitophagy (*PINK1*, p = 0.01; **Figure 8***)* and decrease expression important for autophagosome formation (*ATG7*, p = 0.04; **Figure 8**). (Summarized in **Figure 9**). No significant interaction effects were observed.

## Discussion

At-risk alcohol use promotes systemic inflammation by increasing differentiation of pro-inflammatory immune cells, such as Th1 and Th17 CD4^+^ T cells. These studies sought to determine whether ethanol-mediated changes in CD4^+^ T cell immunometabolism regulate differentiation. Results indicate that 50 mM ethanol, observed in high-risk drinkers and translates to a blood alcohol concentration (BAC) of 0.23 g/dl, shifts differentiation of activated naïve CD4^+^ T cells toward pro-inflammatory Th1 and away from immunosuppressive Treg cells. This shift in phenotype appears to be dependent on ethanol-mediated impaired bioenergetics that is associated with increased Treg mitochondrial content and dysregulation of genes implicated in mitochondrial maintenance and mitophagy.

Functional bioenergetics are crucial for CD4^+^ T cell differentiation. Numerous studies have demonstrated that dysregulation of either glycolysis or oxidative phosphorylation impairs CD4^+^ T cell differentiation (32–37). In this study, the *in vitro* ethanol-mediated dysregulation of CD4^+^ T cell differentiation under Th1-promoting conditions, by increasing Tbet-expressing and decreasing FOXP3-expressing CD4^+^ T cells, recapitulated previously reported literature that *Tbet : FOXP3* ratio was higher in CD4^+^ T cells isolated from the colon of ethanol-administered mice (16). Treg CD4^+^ T cells produce anti-inflammatory cytokines IL-10, IL-35, and TGF-β (48). Decreased IL-10 production promotes Th1 differentiation, as IL-10 is a critical negative regulator of Th1 differentiation (49). Thus, an ethanol-mediated decrease in Treg differentiation could potentially result in decreased anti-inflammatory T cells, anti-inflammatory cytokine production, and promote proinflammatory Th1 differentiation.

Ethanol increased CD4^+^ T cell GLUT1 expression, glucose uptake, and glycolytic rate. GLUT1 is the main glucose transporter expressed by T cells (50). It has been shown that increased GLUT1 expression on activated T cells correlates with increased effector function and IFN-γ production, one of the predominant Th1 cytokines (50). Further, high glycolytic rates are associated with increased permissiveness of HIV viral infection of CD4^+^ T cells (51, 52). This highlights a possible consequence of ethanol-mediated increases in GLUT1-expressing Th1 cells to increase HIV replication in viral reservoirs, and could potentially contribute to the observed increase in viral replication that was observed in SIV-infected chronic binge alcohol (CBA)-administered rhesus macaques (10–13). Our results indicate that inhibition of glycolysis with 2-DG, prevented the ethanol-mediated increase in Th1 differentiation without altering FOXP3-expressing CD4^+^ T cells. These findings agree with previous reports that Tregs do not rely on glycolysis for their differentiation and function (28–30). Thus, our results indicate that ethanol increases Th1 differentiation by promoting glycolysis in differentiated CD4^+^ T cells.

We observed that ethanol decreased CD4^+^ T cell maximal respiration, coupling efficiency, ATP production, and increased proton leak, indicative of impaired mitochondrial function. These changes in mitochondrial respiration were observed in enriched Treg T cells. Treg differentiation is dependent on mitochondrial function, and the ethanol-mediated impairment of oxidative phosphorylation could partially be responsible for the decrease in Treg CD4^+^ T cells caused by ethanol. Ethanol increased mitochondrial volume in enriched Treg cells, without significantly altering Th1 cell mitochondrial content. An increase in mitochondrial content has been associated with an inability to repair dysfunctional mitochondria by autophagy/mitophagy and an increase in cell death (41, 43, 53–55). Also, mitochondrial swelling due to ethanol-induced endoplasmic reticulum stress could be responsible for an increase in mitochondrial volume (56). Based on previous literature (41) and results from this study, we postulate that the increase in mitochondrial volume is due to dysfunctional mitochondrial repair and mitophagy, suggesting an inability to properly eliminate dysfunctional mitochondria.

To explore potential mechanisms underlying increased mitochondrial content, CD4^+^ T cell expression of genes involved in mitophagy was assessed. Ethanol increased expression of *PINK1*, important for mitophagy, and decreased expression of *ATG7*, important for autophagosome formation, within differentiated CD4^+^ T cells. ATG7 is critical for both the survival and development of T cells (57). Further, it was shown that ATG7-deficient T cells had increased mitochondrial content, ROS production, and an imbalanced expression of pro- and anti-apoptotic proteins leading to increased T cell death. We propose that these changes in mitochondrial gene expression may indicate that the ethanol-mediated impairments in mitophagy, specifically autophagosome formation, potentially leads to Treg cell death.

This study has some limitations, as it was performed using immune cells from blood bank donors to identify ethanol’s potential impact on CD4^+^ T cell immunometabolism and differentiation, which no study to date has addressed. Future studies are warranted to translate these findings to people with at-risk alcohol use. This study used only naïve CD4^+^ T cells rather than PBMCs, and has not assessed functional measures including CD4^+^ T cell cytokine production to complement the transcription factor expression. MACS sorting was used to examine CD4^+^ subsets, which only moderately enriched for Tregs and Th1 CD4^+^ subsets, and it was not feasible to obtain homogenous Treg and Th1 populations. Ongoing studies will determine the functional implications of the observed results and whether it mechanistically contributes to increased HIV viral replication. Further, there is a gap in the literature on whether alcohol dysregulates mTOR or AMPK pathways in T cells. mTOR in the absence of alcohol is involved in differentiation of more pro-inflammatory CD4^+^ T cell subsets and AMPK inhibition promotes Treg differentiation (29–32). Our group and others have shown that alcohol-administration impairs mTOR signaling in skeletal muscle and heart (58–60) and AMPK signaling in human alveolar macrophages and heart (61, 62). If alcohol impairs mTOR or AMPK signaling in T cells to the same extent as observed in other tissues, we would expect to see impaired Th1 and Treg differentiation, but currently we only see decreased FOXP3-expressing CD4^+^ T cells (Treg) within our acute *in vitro* ethanol model. Further understanding of these ethanol-related impairments, as well as addressing the limitations of the study outlined above will be addressed in future studies.

Our findings advance our understanding of the mechanisms underlying ethanol-mediated disruption of differentiation of pro- and anti-inflammatory T cells (**Figure 9**). Our study is the first to show that alcohol-mediated shifts in T cell subsets were associated with changes in immune cell bioenergetics. We also show that the observed mitochondrial dysfunction in Tregs was associated with increased mitochondrial content and dysregulated expression of genes implicated in mitophagy and autophagosome formation. Future studies are warranted to systematically investigate whether strategies to maintain immunometabolic homeostasis can ameliorate alcohol-mediated proinflammatory shifts of CD4^+^ T cells. This will provide evidence for potential lifestyle or therapeutic interventions to improve T cell immunometabolism and alleviate risk and progression of autoimmune or viral diseases in people with at-risk alcohol use (63).

## Data Availability Statement

The original contributions presented in the study are included in the article/**Supplementary Material**. Further inquiries can be directed to the corresponding author.

## Author Contributions

PMM performed the experiments; PMM, DL, DW, LS, RS, and PEM provided intellectual discussion. PMM, LS, RS, and PEM designed the experiments. PMM, LS, and RS wrote the manuscript. All authors critically revised and approved the manuscript.

## Funding

This research was supported by the National Institute for Health T32AA007577 and P60AA009803.

## Conflict of Interest

The authors declare that the research was conducted in the absence of any commercial or financial relationships that could be construed as a potential conflict of interest.

## Publisher’s Note

All claims expressed in this article are solely those of the authors and do not necessarily represent those of their affiliated organizations, or those of the publisher, the editors and the reviewers. Any product that may be evaluated in this article, or claim that may be made by its manufacturer, is not guaranteed or endorsed by the publisher.

## Supplementary Material

Supplementary Figure 1 IL-12 promotes FOXP3 expression similar to TGF-beta + IL-2 in CD3/CD28 stimulated CD4^+^ T cells.

Supplementary Figure 2 Gating strategy used for assessment of master transcription factor expression in CD4^+^ T cells.

Supplementary Figure 3 Moderate enrichment of CD25+FOXP3+ and CD25-Tbet+ CD4 T cells by MACS sorting. **(A)** CD4+CD25+ T cells were enriched with FOXP3+ Cells. **(B)** CD4+CD25- T cells were enriched with Tbet+ Cells.

Supplementary Figure 4 Gating strategy used for assessment of 2-NBDG uptake in CD4^+^ T cells.

Supplementary Figure 5 Gating strategy used for assessment of Mitotracker Deep Red mitochondrial stain in CD4^+^ T cells.

Supplementary Figure 6 Bioenergetic Health Index (BHI) and Oxygen Consumption Rate (OCR)/Extracellular Acidification Rate (ECAR) of enriched Treg and Th1 subsets. **(A)** There were no significant differences observed in BHI between DIFF and DIFF + EtOH of enriched Treg or Th1 subsets. **(B)** There were no significant differences observed in OCR/ECAR between DIFF and DIFF + EtOH of enriched Treg of Th1 subsets. Paired T test.
