# Supplementary figures and images for: Alcohol Impairs Immunometabolism and Promotes Naïve T Cell Differentiation to Pro-Inflammatory Th1 CD4+ T Cells
Source: Front Immunol. 2022 May 12;13:839390. doi: 10.3389/fimmu.2022.839390 (PMC9133564; doi:10.3389/fimmu.2022.839390)

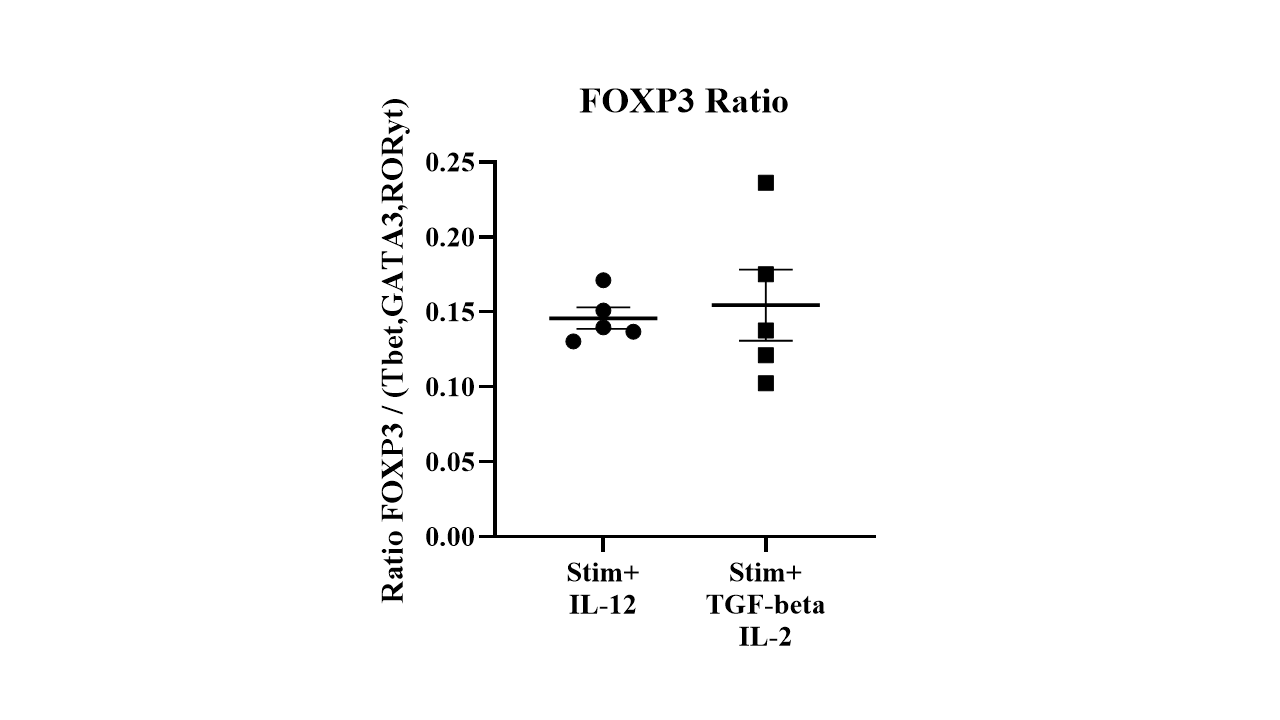

Supplement: Supplementary Figure 1 — IL-12 promotes FOXP3 expression similar to TGF-beta + IL-2 in CD3/CD28 stimulated CD4+ T cells. [file Image_1.tif]

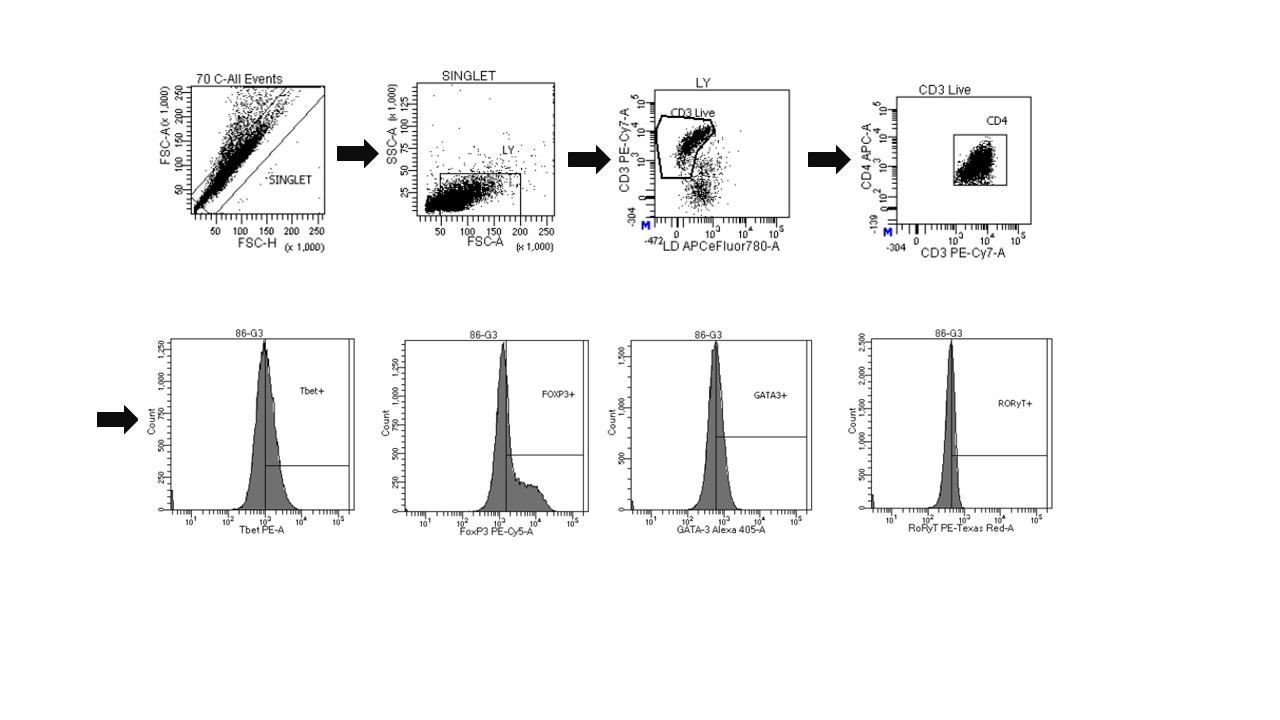

Supplement: Supplementary Figure 2 — Gating strategy used for assessment of master transcription factor expression in CD4+ T cells. [file Image_2.tif]

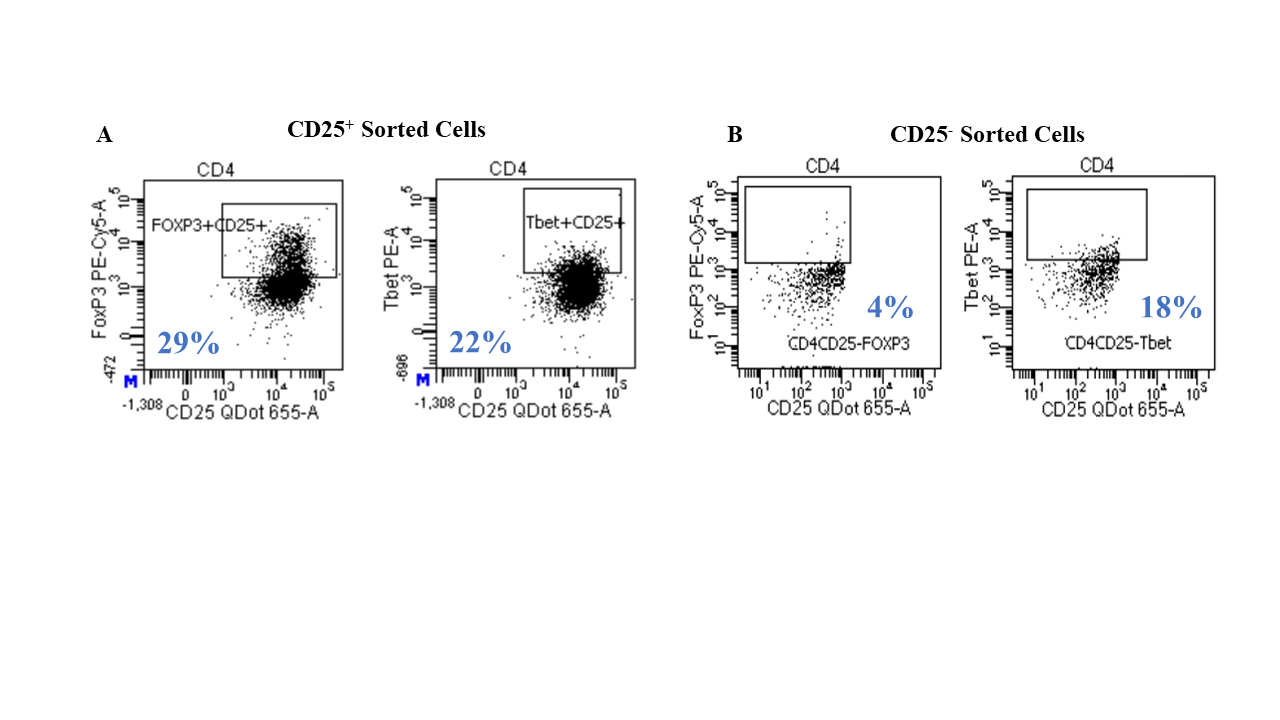

Supplement: Supplementary Figure 3 — Moderate enrichment of CD25+FOXP3+ and CD25-Tbet+ CD4 T cells by MACS sorting. (A) CD4+CD25+ T cells were enriched with FOXP3+ Cells. (B) CD4+CD25- T cells were enriched with Tbet+ Cells. [file Image_3.tif]

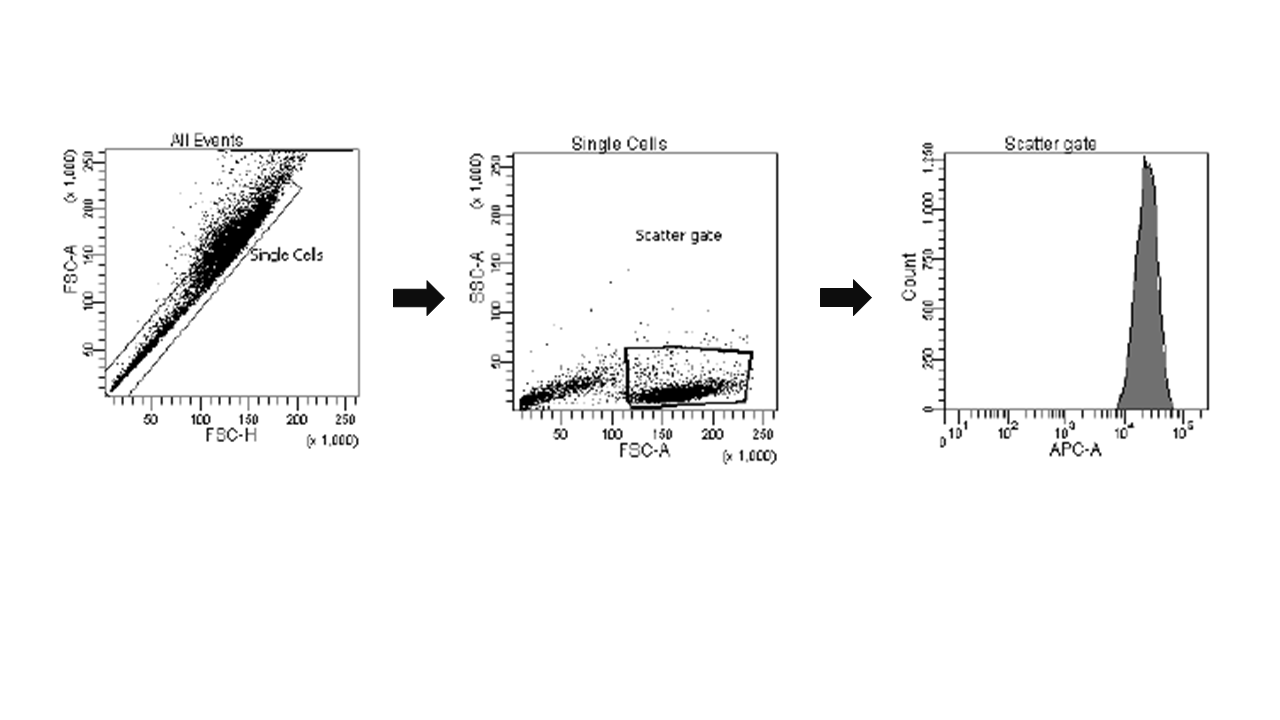

Supplement: Supplementary Figure 4 — Gating strategy used for assessment of 2-NBDG uptake in CD4+ T cells. [file Image_4.tif]

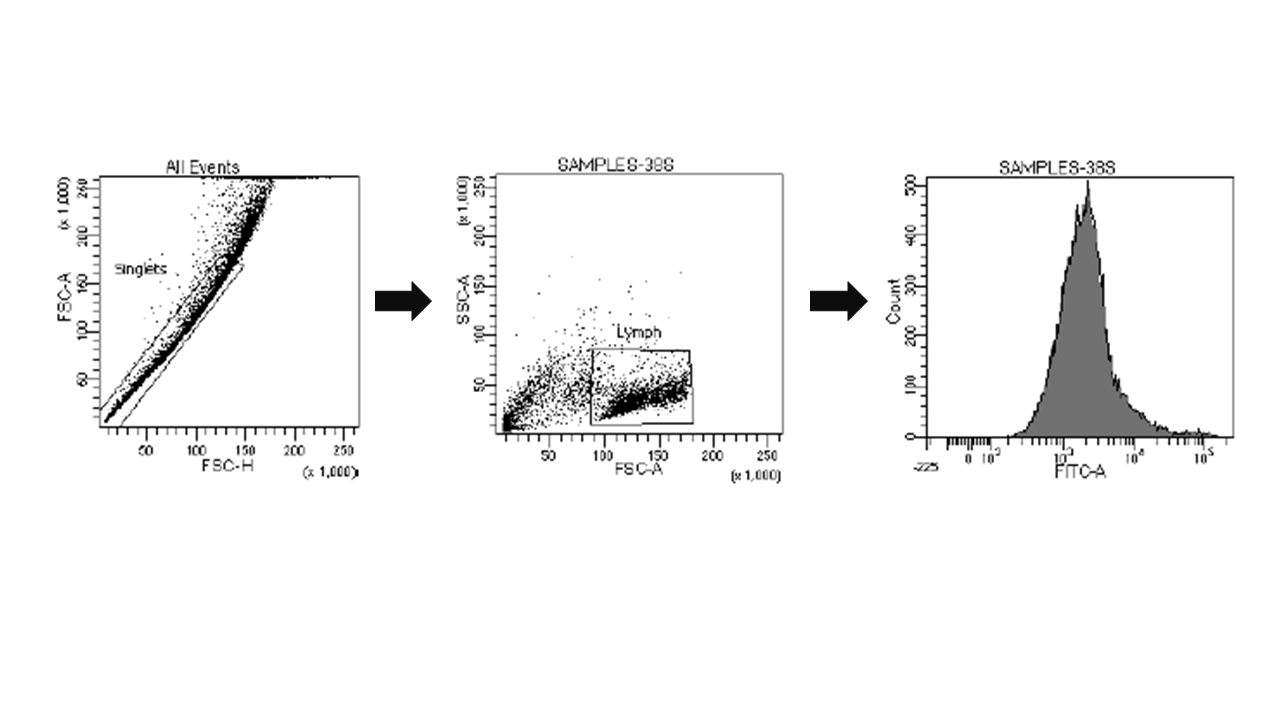

Supplement: Supplementary Figure 5 — Gating strategy used for assessment of Mitotracker Deep Red mitochondrial stain in CD4+ T cells. [file Image_5.tif]

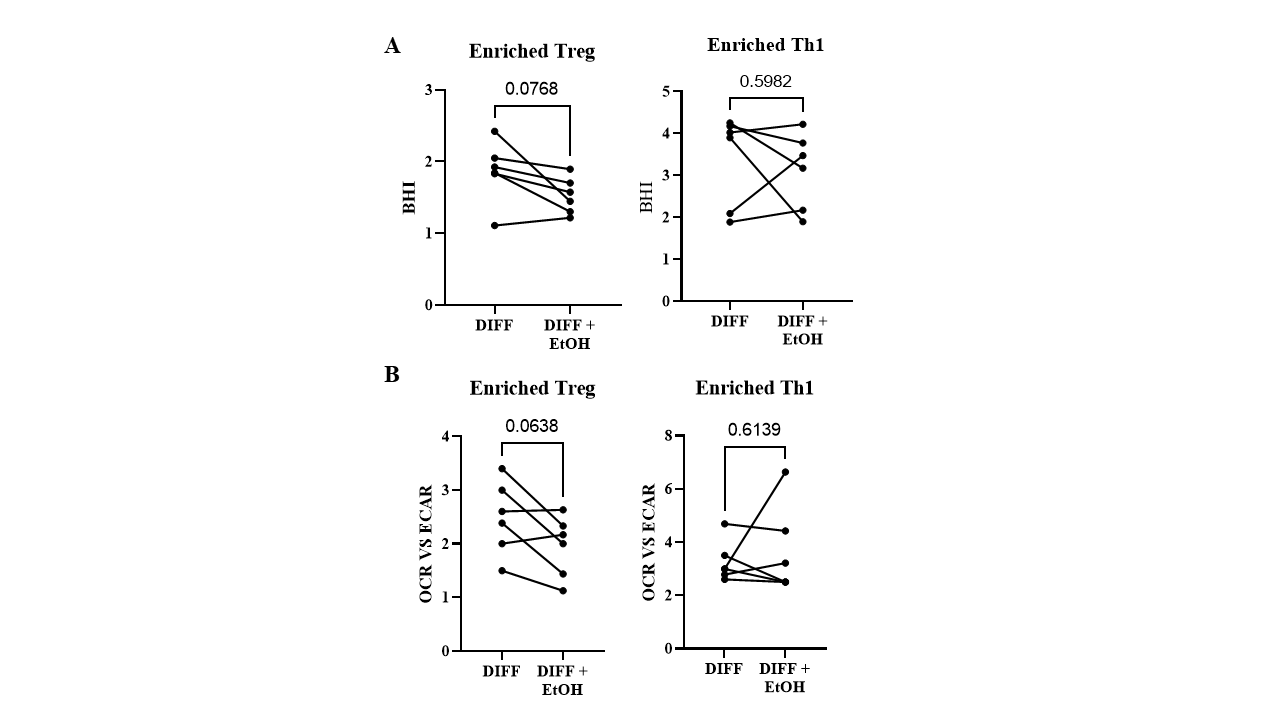

Supplement: Supplementary Figure 6 — Bioenergetic Health Index (BHI) and Oxygen Consumption Rate (OCR)/Extracellular Acidification Rate (ECAR) of enriched Treg and Th1 subsets. (A) There were no significant differences observed in BHI between DIFF and DIFF + EtOH of enriched Treg or Th1 subsets. (B) There were no significant differences observed in OCR/ECAR between DIFF and DIFF + EtOH of enriched Treg of Th1 subsets. Paired T test. [file Image_6.tif]
